# Concurrent radiotherapy and weekly chemotherapy of 5-fluorouracil and platinum agents for postoperative locoregional recurrence of oesophageal squamous cell carcinoma

**DOI:** 10.1038/srep08071

**Published:** 2015-01-28

**Authors:** Wen-Wen Zhang, Yu-Jia Zhu, Han Yang, Qiao-Xuan Wang, Xiao-Hui Wang, Wei-Wei Xiao, Qiao-Qiao Li, Meng-Zhong Liu, Yong-Hong Hu

**Affiliations:** 1 State Key Laboratory of Oncology in South China, Sun Yat-sen University Cancer Center, Guangzhou, Guangdong, P.R. China; 2 Department of Radiation Oncology, Sun Yat-sen University Cancer Center, Guangzhou, Guangdong, P.R. China; 3 Department of Thoracic Surgery, Sun Yat-sen University Cancer Center, Guangzhou, Guangdong, P.R. China; 4 Guangdong Esophogeal Cancer Research Institute, Guangzhou, Guangdong, P.R. China

## Abstract

The most optimal management for postoperative locoregional recurrence of oesophageal squamous cell carcinoma is still controversial. Several studies have reported the feasibility and efficacy of concurrent chemoradiotherapy (CCRT), mostly with three-weekly or four-weekly schedule of chemotherapy. However, treatment compliance was not quite satisfactory, probably due to treatment-related toxicities. Since CCRT with weekly chemotherapy regimens have demonstrated a favorable toxicity profile as well as promising survival in certain types of cancer, we aimed to evaluate the efficacy and toxicity of radiotherapy concurrently with weekly chemotherapy with 5-fluorouracil (5-FU) and platinum agents for patients with postoperative locoregional recurrence of oesophageal squamous cell carcinoma in our center. Twenty-seven consecutive patients who were diagnosed with postoperative locoregional recurrence of oesophageal squamous cell carcinoma and received CCRT with weekly chemotherapy of 5-FU and platinum agents were retrospectively analyzed. Our data showed that the present protocol of radiotherapy combined concurrently with weekly chemotherapy of 5-FU and platinum agents was a safe and effective salvage treatment for postoperative locoregional recurrence of oesophageal squamous cell carcinoma.

Oesophageal cancer is one of the most fatal malignancies worldwide. Unlike western countries, the most common histological subtype of oesophageal cancer in China is squamous cell carcinoma^1^. Surgical resection has been the primary treatment for patients with resectable oesophageal cancer. However, even after radical surgery, the overall survival (OS) remains poor and locoregional recurrence has been the major pattern of failure^2^,^3^,^4^,^5^,^6^,^7^,^8^,^9^,^10^. The most optimal management for postoperative locoregional recurrence of oesophageal squamous cell carcinoma is still controversial. Studies on surgical resection, chemoradiotherapy, or surgery plus chemoradiotherapy in the management of locoregional recurrent oesophageal cancer reported similar results with regard to survival and local control^11^. Despite this, chemoradiotherapy showed beneficial effects on symptomatic control and might improve long-term survival in selected patients^12^,^13^. To now, several studies have reported the feasibility and efficacy of concurrent chemoradiotherapy in postoperatively recurrent oesophageal squamous cell carcinoma, mostly with three-weekly or four-weekly schedule of chemotherapy^14^,^15^,^16^. However, nearly one quarter of patients required a reduction in or omission of the second cycle of chemotherapy^16^. In certain types of cancer, such as epithelial ovarian cancer, cervical cancer and non-small cell lung cancer, promising activity and a favorable toxicity profile have been reported when using weekly chemotherapy regimens^17^,^18^,^19^,^20^,^21^,^22^,^23^. Additionally, in oesophageal squamous cell carcinoma, a recently published phase II study has reported the feasibility and efficacy of CCRT with weekly cisplatin-only regimen in patients with postoperative mediastinal lymph node metastasis^24^. In this retrospective study, we evaluated the efficacy and toxicity of radiotherapy concurrently with weekly chemotherapy of 5-FU and platinum agents in patients with postoperative locoregional recurrence of oesophageal squamous cell carcinoma.

## Methods

### Study population

We reviewed the documents of patients who were diagnosed with postoperative locoregional recurrence of oesophageal squamous cell carcinoma and received radiotherapy in our center between January 2009 and December 2013. Inclusion criteria of this study included: (1) R0 resection for primary oesophageal squamous cell carcinoma with two-incision oesophagectomy (Ivor Lewis approach)^25^ or three-incision (right thoracotomy, midline laparotomy and left cervical incisions) oesophagectomy with cervical oesophagogastric anastomosis; (2) absence of previous thoracic radiotherapy; (3) cervical and/or thoracic postoperative recurrence (biopsy proven or positron emission tomography/computed tomography (PET/CT) proven or follow-up computed tomography (CT) showed progression of disease); (4) absence of distant metastasis at recurrence; (5) Eastern Cooperative Oncology Group (ECOG) performance status (PS) ≤ 2; (6) concurrent three dimensional conformal radiotherapy (3D-CRT) or intensity modulated radiotherapy (IMRT) and weekly chemotherapy of 5-FU plus cisplatin or nedaplatin as salvage treatment; (7) regular follow-up after completion of treatment. A total of 27 patients met the criteria and were included in this study.

Baseline evaluations generally included: a complete medical history and physical examination; complete blood count and serum chemistry profile; electrocardiogram; pulmonary function test; barium-swallow examination; contrast-enhanced CT scan of the neck, chest and upper abdomen or who-body PET/CT; and endoscopic ultrasound. The 7th edition of American Joint Committee on Cancer (AJCC) staging system for oesophageal cancer released in 2010 was used to restage the primary diseases after radical surgery.

Written informed consent was obtained from every patient for the treatment, publication of this report and all accompanying images.

### Chemotherapy regimen

All of the 27 patients received CCRT as salvage treatment in our institution. Fourteen patients were treated with 4 cycles of weekly cisplatin and 5-FU (CF regimen: cisplatin 25 mg/m^2^ on day 1, 5-FU 300 mg/d on day 1–3, repeated weekly for 4 weeks) concurrently with radiotherapy. Thirteen patients were treated with 4 cycles of weekly nedaplatin and 5-FU (NF regimen: nedaplatin 25 mg/m^2^ on day 1, 5-FU 300 mg/d on day 1–3, repeated weekly for 4 weeks) concurrently with radiotherapy.

### Radiotherapy administration

With the patient in supine position, a cradle for immobilization was made with vacuum. Individual patient was scanned from the axis (C2) to the second lumbar vertebra (L2) level to cover the entire neck, lung, esophagus and celiac lymph node regions. CT scan was performed with 0.5 cm thick slices. Briefly, the gross tumor volume (GTV) consisted of recurrent lesion shown by PET/CT or subsequent CT scans; tumor lesions discovered under endoscopy but not seen on PET/CT or CT were also included in the GTV. Lymph nodes showing at least one of the following features on CT were defined as positive nodes: short axis ≥10 mm, distribution in a cluster of lymph nodes, infiltrative margin, or central necrosis. Lymph nodes that demonstrated uptake on the PET/CT scan were also included in the GTV, regardless of size. The clinical target volume (CTV) comprised the anastomosis, supraclavicular, and station 1–5 and 7 lymph nodes. PTV1 was defined as the GTV plus a margin of 5 mm and PTV2 was defined as the CTV plus a margin of 5 mm in all directions, respectively. 3D-CRT treatment plans were calculated by Pinnacle planning system and IMRT treatment plans were calculated by Monacle planning system. All patients were treated with a 6-MV linear accelerator. The prescribed dose was generally 50–60 Gy to PTV1 and 46–50 Gy to PTV2. Dose constraints for critical organs were: spinal cord <45 Gy, mean lung dose <17 Gy and lung dose V20 < 30%.

### Treatment outcome and toxicity

Patients were evaluated every week during therapy. Complete blood count, serum chemistry profile and barium-swallow examination were repeated every week during CCRT. After treatment, patients were followed up at 2 month, then every 3 months for the first year, every 6 months for 2 years and then annually thereafter or when clinically indicated. Medical history, physical examination, barium-swallow examination, and contrast-enhanced CT scan of the neck, chest and upper abdomen were required for each follow-up visit. Endoscopy, brain magnetic resonance imaging (MRI), radionuclide bone scanning and PET/CT depended on the discretion of the treating physicians.

The overall survival (OS) was defined as time from start of CCRT to death of any cause. The progression free survival (PFS) was defined as time from start of CCRT to progression, relapse, or death of any cause. Locoregional control (LRC) was defined as time from start of CCRT to local and/or regional progression or relapse. Tumor response was defined according to the criteria of Response Evaluation Criteria in Solid Tumors version 1.1 (RECIST 1.1). A senior radiologist together with a radiation oncologist performed evaluation of tumor response about 2 month after the end of CCRT. Acute toxicity was defined as treatment-related toxicity happened within 6 months after the beginning of CCRT and evaluated according to the Common Terminology Criteria for Adverse Events version 3.0 (CTCAE 3.0). Patterns of failure were defined as the first site of failure. Locoregional failure included the primary tumor and regional lymph nodes. Distant failure included any site beyond the primary tumor and regional lymph nodes.

### Statistical analysis

SPSS software for Windows, version 19.0 was utilized to perform statistical analysis. Kaplan-Meier method and multivariable forward stepwise Cox proportional hazards analysis were performed for survival analysis. A two-sided *P* value of <0.05 was considered significant.

## Results

### Patient characteristics

Baseline characteristics of the 27 patients are listed in Table 1. The cohort had a male predominance (74.1%). The median age was 55 years (ranging from 42 to 75 years). Primary tumors were mostly located in the middle thoracic esophagus (77.8%). Ten (37.0%) patients received two-incision oesophagectomy while 17 (63.0%) received three-incision oesophagectomy. In regard to postoperative stage, 2 (7.4%) patients had stage I disease, 10 (37.0%) patients had stage II, and 15 (55.6%) patients had stage III. After the surgery, 13 (51.9%) patients received adjuvant chemotherapy. All the patients received R0 resection and regular follow-up after completion of treatment. The median time to recurrence after initial treatment was 17 months (ranging from 4 to 132 months). Nine (33.3%) patients had anastomotic recurrence (AR) with or without locoregional lymphadenopathy, and 18 (66.7%) patients had mediastinal and/or supraclavicular lymph node recurrence (LR). The most common involved lymph node stations were 4R (9/27, 33.3%), 2R (8/27, 29.6%), 4L (6/27, 22.2%) and 7 (6/27, 22.2%).

### Treatment

Treatment information for locoregional recurrent disease is shown in Table 2. All patients were treated with CCRT, including 14 patients receiving weekly CF regimen and 13 patients receiving weekly NF regimen as concurrent chemotherapy. Seventeen (63.0%) patients had 3D-CRT, while 10 (37.0%) had IMRT. The median radiation dose was 56.0 Gy, ranging from 46.0 Gy to 60.0 Gy. All patients received 4 cycles of concurrent chemotherapy. And almost all patients completed the planned radiotherapy except for one patient, who refused to continue the treatment for personal reasons at a radiation dose of 46 Gy.

### Treatment outcome

**Figure 1:**
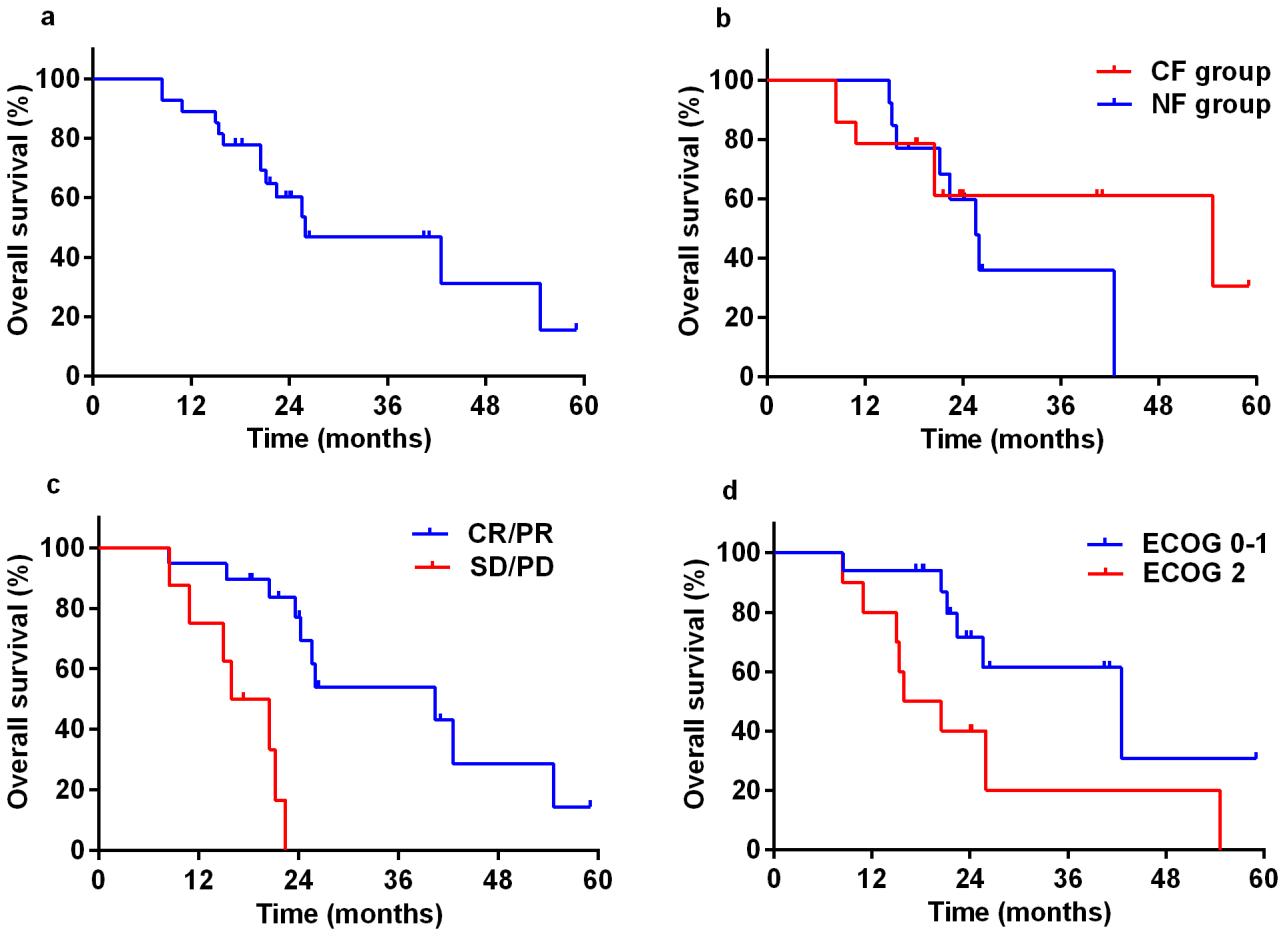
Overall survival of (a) all patients (n = 27), (b) patients treated with radiotherapy combined concurrently with weekly chemotherapy of cisplatin plus 5-FU (CF group, n = 14) or nedaplatin plus 5-FU (NF group, n = 13), (c) patients achieved complete/partial remission (CR/PR, n = 19) or stable/progressive disease (SD/PD, n = 8), and (d) patients with Eastern Cooperative Oncology Group Performance Status Scale (ECOG PS) of 0–1 (n = 17) or 2 (n = 10).

After treatment completion, 19 (70.4%) patients achieved complete remission (CR) or partial remission (PR) (Table 2). The median follow-up time was 22.4 months (range, 8.4–59.1 months) for all patients. At the time of this analysis, 14 (51.9%) patients died of oesophageal cancer. No treatment-related death occured. Thirteen (48.1%) patients were alive with (n = 11) or without (n = 2) disease. As shown in Figure 1a, the 1-year and 2-year OS for all patients were 88.9% and 60.2%, respectively, with a median survival time of 26.0 months (95% CI, 10.5–41.4 months). The 1-year and 2-year PFS were 66.7% and 33.9%, respectively, with a median PFS time of 16.4 months (95% CI, 13.4–19.4 months). The 1-year and 2-year LRC were 70.4% and 46.1%, respectively.

Survival predictors were analyzed using univariate analysis followed by Cox regression model. (Table 3). Patients receiving CF or NF as concurrent chemotherapy regimens had similar survival (2-year OS: 61.6% vs. 59.8%, P = 0.440, Figure 1b). Tumor response, ECOG PS, and operation type were entered into Cox multivariate regression model as they showed a *P* value <0.20 in the univariate analysis. Tumor response (HR = 0.065, 95% CI, 0.014–0.301, *P* = 0.000) and ECOG PS (HR = 4.392, 95% CI, 1.352–14.269, *P* = 0.014) were found to be independent predictors for overall survival (Table 3, Figure 1c and 1d).

### Patterns of failure

Treatment failure was observed in 17 (63.0%) patients during the follow-up period. Ten (37.0%) patients experienced locoregional failure only, four (14.8%) had distant failure only, and three (11.1%) had both locoregional and distant failure.

### Treatment-related toxicities

As shown in Table 4, all toxicities were tolerable and reversible. Most treatment-related toxicities were grade 1 to 2. Grade ≥3 leukocytopenia occurred in 6 (22.2%) patients, with 3 (21.4%) in the CF group and 3 (23.1%) in the NF group, respectively (*P* = 1.000). Other toxicities of grade ≥3 included thrombocytopenia (one patient in the NF group), anemia (one in the NF group), and nausea and vomiting (one in the CF group and one in the NF group). No grade ≥3 oesophagitis was observed. The incidence and severity of all toxicities were comparable between the CF and NF groups. There was no radiation-induced lung injury or treatment-related death.

## Discussion

To our knowledge, this is the first study investigating the efficacy and safety of concurrent radiotherapy and chemotherapy with weekly administration of 5-FU and platinum agents for patients with postoperative locoregional recurrence of oesophageal squamous cell carcinoma. Our results show that, with this weekly schedule, patients achieved a high overall response rate (CR + PR, 70.4%), as well as promising survival (2-year OS, 60.2%). Moreover, this treatment strategy was well tolerated, as the incidence of grade ≥3 toxicities was quite low and no treatment-related death was observed. We also found that, patients who responded (CR/PR) to this salvage treatment or with a good ECOG performance status (0–1) had a better prognosis.

There have been a few studies on the efficacy of radiotherapy with or without chemotherapy as salvage treatment for postoperative locoregional recurrence of oesophageal cancer. Studies with more than 20 patients are listed in Table 5^11^,^12^,^13^,^14^,^15^,^16^,^24^,^26^,^27^,^28^,^29^,^30^,^31^. In those studies, the reported response rates were generally high, mostly above 70%. However, the survival showed great variation, with the median survival time ranging from 7 to 43 months, the 1-year OS ranging from 33.8% to 85.7%, and the 2-year OS ranging from 15% to 51.3%. Several factors might account for this discrepancy. Firstly, as those studies were mostly small sample-sized retrospective studies, selection bias could exist; secondly, some studies included patients receiving radiotherapy alone^12^,^13^,^28^,^29^,^31^; thirdly, even within patients receiving chemoradiotherapy, the chemotherapy regimens were diverse; fourthly, the radiotherapy techniques also differed, as some studies used 2D conventional radiotherapy while others used 3D-CRT; finally, the treatment target volume definition and irradiation dose were also various. All of those factors mentioned above could influence the clinical outcome remarkably. In our study, a total of 27 patients were treated for postoperative locoregional recurrence with 3D-CRT or IMRT (median dose 56 Gy, range, 46–60 Gy) combined with concurrent chemotherapy (14 patients with weekly CF regimen, while the other 13 patients with weekly NF regimen). The overall response rate was 70.4%. The 1-year and 2-year OS rates were 88.9% and 60.2%, respectively, with a median survival time of 26.0 months for all patients. Compared with previous studies listed in Table 5, the clinical outcome in our study was generally favorable. Although our results should be interpreted with caution for the small sample size and short observation period, median survival time of 26 month and 2-year OS rate of 60.2% can be looked as encouraging, indicating the efficacy of weekly schedule of 5-FU and platinum agents for postoperative locoregional recurrence of oesophageal squamous cell carcinoma.

Among those studies, one which we would like to focus on was the study reported by Bao Y et al.^14^. In this study, a total of 83 patients were treated for postoperative locoregional recurrence of oesophageal squamous cell carcinoma with 3D-CRT (median dose 60 Gy, range, 56–68 Gy) combined with concurrent chemotherapy (42 patients with four-weekly CF regimen, 26 patients with four-weekly docetaxel plus cisplatin (TP) regimen, and 15 patients with weekly TP regimen). The overall response rate was 75.9%. The 3-year OS rate was 51.8% with a median survival time of 43.0 months. Compared with this result, the overall response rate in our study was comparable, while the median survival time was much shorter (26 months vs. 43 months). A possible explanation was the differences in radiation dose and chemotherapy regimens. The radiation dose of our study was lower than that of Bao's study, with a median dose of 56 Gy (range, 46–60 Gy) vs. 60 Gy (range, 56–68 Gy). Although the optimal radiation dose for locoregional recurrence has not been established for oesophageal squamous cell carcinoma, some studies demonstrated that a high radiation dose might yield better prognosis^12^,^30^,^31^. Zhang et al reported that a radiation dose of more than 60 Gy was associated with a trend toward a better OS^30^. In our study, only 12 (44.4%) patients received a total radiation dose of 60 Gy, and no patient received more than 60 Gy, which might partially attribute to the relatively dismal outcome compared with that in Bao's study. Another reason was that nearly half of the patients in Bao's study received TP regimen concurrently with radiotherapy^14^. Although no sufficient evidence has been established for TP regimen in treatment of postoperative recurrence of oesophageal squamous cell carcinoma, the Swiss Group for Clinical Cancer Research (SAKK) has reported a preoperative induction schedule comprising TP regimen followed by CCRT to be effective and feasible, with a 3-year OS of 53%^32^. Likewise, some studies have examined the use of docetaxel and platinum compounds as neoadjuvant chemotherapy and radiosensitizers for oesophageal cancer, reporting high pathological CR rates^33^,^34^. In Bao's study, a survival advantage for patients treated with TP regimen compared with patients treated with CF regimen (3 year OS, 59.2% vs. 43.3%, *p* = 0.010) was reported. The adoption of TP regimen as concurrent chemotherapy might contribute to a favorable survival in Bao's study and need to be further evaluated.

The treatment-related toxicity and compliance are always important issues for patients who received CCRT. As shown in our study, the most frequent acute toxicity was leukocytopenia, grade 3–4 in 6 (22.2%) patients. Grade 3 thrombocytopenia was only observed in one (3.7%) patient in the NF group. Other grade ≥3 toxicities were only observed in two (7.4%) patients, with grade 3 anemia in one (3.7%) patient and grade 3 nausea and vomiting in one (3.7%) patient. No life-threatening toxicity or treatment-related death was observed. Treatment compliance was also good, as only one (3.7%) patient had interruption of treatment. In a phase II study on CCRT with three-weekly NF regimen chemotherapy, the reported grade ≥3 toxicities were as follows: grade 3 leukocytopenia in 9 (30%) patients, grade 4 thrombocytopenia in one (3.3%) patient, grade 3–4 diarrhea in two (6.7%) patients, and grade 3 heartburn or mucositis in one (3.3%) patient. The rate of completion of the treatment in this study was 76.7%^16^. As reported by Bao Y et al., the most frequent toxicity observed was grade 3 vomiting, which occurred in 18 (21.7%) patients. Other grade ≥3 toxicities were as follows: grade 3 neutropenia in 17 (20.5%) patients and grade 3 oesophagitis in 5 (6.0%) patients^14^. Despite the similarly high incidence of hematotoxicity, the incidence of grade ≥3 gastrointestinal toxicity and oesophagitis were relatively low in our study, compared with the previous two studies^14^,^16^. Several factors may account for this difference, including the different chemotherapy regimens and schedule, the incorporation of IMRT, and the relatively low radiation dose that were used in our study.

Several limitations existed in our study, including the relatively small sample size, the retrospective nature, and the inadequate follow-up period. In addition, the radiation dose and radiotherapy techniques were sort of heterogenous.

## Conclusion

The results of the present retrospective analysis indicate that the present protocol of radiotherapy combined concurrently with weekly chemotherapy of 5-FU and platinum agents was a safe and effective salvage treatment for postoperative locoregional recurrence of oesophageal squamous cell carcinoma. However, as this is a small sample-sized retrospective study, further prospective trial with a larger sample size is warranted.

## Author Contributions

Y.H.H. is the guarantor of the manuscript and contributed to the concept and design. W.W.Z. contributed to analysis and interpretation of the data and the drafting and revision of the manuscript. Y.J.Z. contributed to the concept and design, clinical data collection, and editing and revision of the manuscript. H.Y. contributed to clinical data collection, and editing and revision of the manuscript. Q.X.W. and X.H.W. participated in editing and revision of the manuscript. W.W.X., Q.Q.L. and M.Z.L. performed the chemoradiotherapy and the follow-up. All authors read and approved the final manuscript.

## Figures and Tables

**Table 1:** Baseline clinical characteristics of patients with postoperative locoregional recurrence of oesophageal squamous cell carcinoma

Variable	*n* (%)
**Gender**	
Male	20 (74.1)
Female	7 (25.9)
**Age** (year)	
Median (range)	55 (42–75)
<60	20 (74.1)
≥60	7 (25.9)
**ECOG PS**	
0–1	17 (63.0)
2	10 (37.0)
**BMI (**kg/m^2^**)**	
<18.5	8 (29.6)
≥18.5	19 (70.4)
**CCI**	
0	17 (63.0)
1–2	10 (37.0)
**Smoking status**	
Former or current smoker	15 (55.6)
Non-smoker	12 (44.4)
**Drinking**	
No	15 (55.6)
Yes	12 (44.4)
**Primary tumor location**	
Upper	3 (11.1)
Middle	21 (77.8)
Lower	3 (11.1)
**TNM Stage of primary tumor**^a^	
Ia, Ib	2 (7.4)
IIa, IIb	10 (37.0)
IIIa, IIIb	15 (55.6)
**Histology of primary tumor**	
Grade 1–2	23 (85.2)
Grade 3–4	4 (14.8)
**Primary radical surgery**	
Two-incision oesophagectomy	10 (37.0)
Three-incision oesophagectomy	17 (63.0)
**Adjuvant chemotherapy after surgery**	
No	14 (48.1)
Yes	13 (51.9)
**Time to recurrence after initial treatment**	
Median (range, month)	17 (4–132)
≥1 year	18 (66.7)
<1 year	9 (33.3)
**Recurrence site**	
Anastomosis with/without lymphadenopathy	9 (33.3)
Supraclavicular and/or regional lymph node	18 (66.7)

*Abbreviations:* BMI = body mass index; CCI = Charlson comorbidity index; ECOG PS = the Eastern Cooperative Oncology Group Performance Status Scale;

^a^TNM stage was according to the 7^th^ edition of the American Joint Commission on Cancer (AJCC) staging system.

**Table 2:** Treatment information for locoregional recurrent disease and tumor response (*n* = 27)

Variable	*n* (%)
**Radiotherapy techniques**	
3D-CRT	17 (63.0)
IMRT	10 (37.0)
**Radiation dose** (Gy)	
Median (range)	56.0 (46.0–60.0)
**Concurrent chemotherapy**	
Cisplatin + 5-Fu	14 (51.9)
Nedaplatin + 5-Fu	13 (48.1)
**Tumor response**^a^	
CR	4 (14.8)
PR	15 (55.6)
SD	3 (11.1)
PD	5 (18.5)

*Abbreviations:* 3D-CRT = three-dimensional conformal radiotherapy; CR = complete remission; IMRT = intensity-modulated radiotherapy; PD = progressive disease; PR = partial remission; SD = stable disease;

^a^Tumor response was defined according to the criteria of Response Evaluation Criteria in Solid Tumors version 1.1 (RECIST 1.1).

**Table 3:** Univariate and multivariate analysis of prognostic factors for overall survival (*n* = 27)

	Univariate analysis	Multivariate analysis	
Variable	*P* value	*P* value	HR (95%CI)
Gender (male vs. female)	0.783		
Age (<60 years vs. ≥60 years)	0.338		
BMI (<18.5 kg/m^2^ vs. ≥18.5 kg/m^2^)	0.783		
ECOG PS (0 – 1 vs.2)^b^	0.036	0.014	4.392 (1.352–14.269)
CCI (0 vs. 1 – 2)	0.897		
Location of primary tumor (upper vs. middle vs. lower)	0.222		
Operation (Two-incision vs. Three-incision)^b^	0.192	0.855	
Postoperative Stage (I – II vs. III)^a^	0.566		
Adjuvant chemotherapy (yes vs. no)	0.834		
Recurrence Time (≥1 year vs. <1 year)	0.684		
Recurrence site (AR vs. LR)	0.509		
Radiation techniques (3D-CRT vs. IMRT)	0.209		
Radiation dose (≥56 Gy vs. <56 Gy)	0.274		
Chemotherapy (CF vs. NF)	0.440		
Tumor Response (CR/PR vs. SD/PD)^b^	0.000	0.000	0.065 (0.014–0.301)

*Abbreviations:* 3D-CRT = three-dimensional conformal radiotherapy; AR = anastomosis with/without lymphadenopathy; BMI = body mass index; CCI = Charlson comorbidity index; CF = cisplatin + 5-Fu; CI = confidence interval; CR = complete remission; ECOG PS = Eastern Cooperative Oncology Group Performance Status Scale; HR = Hazard ratio; IMRT = intensity-modulated radiotherapy; LR = supraclavicular and/or regional lymph node; NF = nedaplatin + 5-Fu; PD = progressive disease; PR = partial remission; SD = stable disease;

^a^TNM stage was according to the 7^th^ edition of the American Joint Commission on Cancer (AJCC) staging system.

^b^Tumor response, ECOG PS, and operation type were selected for further multivariate analysis as they showed a *P* value <0.20 in univariate analysis.

**Table 4:** Incidence of acute treatment-related toxicities^a^ (n = 27)

	All patients	CF group (n = 14)	NF group (n = 13)	
	*n* (%)	*n* (%)	*n* (%)	*P* value
Leukocytopenia				
Grade 0	4 (14.8)	2 (14.3)	2 (15.4)	1.000
Grade 1 – 2	17 (63.0)	9 (64.3)	8 (61.5)	
Grade 3 – 4	6 (22.2)	3 (21.4)	3 (23.1)	
Thrombocytopenia				
Grade 0	13 (48.1)	9 (64.3)	4 (30.8)	.174
Grade 1 – 2	13 (48.1)	5 (35.7)	8 (61.5)	
Grade 3	1 (3.7)	0 (0.0)	1 (7.7)	
Anemia				
Grade 0	6 (22.2)	4 (28.6)	2 (15.4)	.648
Grade 1 – 2	20 (74.1)	10 (71.4)	10 (76.9)	
Grade 3	1 (3.7)	0 (0.0)	1 (7.7)	
Nausea and vomiting				
Grade 0	0 (0.0)	0 (0.0)	0 (0.0)	1.000
Grade 1 – 2	26 (96.3)	13 (92.9)	13 (100.0)	
Grade 3	1 (3.7)	1 (7.1)	0 (0.0)	
Oesophagitis				
Grade 0	21 (77.8)	10 (71.4)	11 (84.6)	0.648
Grade 1 – 2	6 (22.2)	4 (28.6)	2 (15.4)	
Liver enzyme elevation				
Grade 0	21 (77.8)	12 (85.7)	9 (69.2)	0.385
Grade 1 – 2	6 (22.2)	2 (14.3)	4 (30.8)	

^a^Acute toxicities were evaluated according to the Common Terminology Criteria for Adverse Events version 3.0 (CTCAE 3.0).

**Table 5:** Previous studies on radiotherapy with or without chemotherapy as salvage treatment for patients with postoperative locoregional recurrence of oesophageal squamous cell carcinoma

Study	*n*	Treatment (*n*)	ORR	MST (month)	1 year OS	2 year OS	3 year OS
Raoul, J. L., 1995^13^	31^a^	RT + CT	65%	<12	47.1%	17.1%	4.3%
Nemoto, K., 2001^29^	33	RT alone (21)	91%	7	33%	15%	12%
		RT + CT (CF) (12)					
Shioyama, Y., 2007^12^	82	RT alone (52)	78%	7	/	22%	/
		RT + CT (30)					
Nakamura, T., 2008^11^	22	RT + CT	82%	20.3	/	/	26.6%
Lu, J. C., 2010^28^	73	RT alone (42)	76%	9	33.8%	/	0.0%
		CCRT with CF (31)	97%	17	62.5%		10.5%
Maruyama, K., 2011^27^	23	RT + CT	/	13	52%	31%	/
Jingu, K., 2012^26^,^16^	30	CCRT with three-weekly NF	73.3%	21	60.6%	/	38.4%
Bao, Y., 2012^14^	83	CCRT (3D-CRT)	75.9%	43	/	/	51.8%
Zhang, J., 2012^30^	50	CCRT with four-weekly CF (22)	72.7%	9.8	56%	/	14%
		CCRT with three-weekly TP (28)	71.4%	16.3			
Fakhrian, K., 2012^31^	54^b^	RT alone (18)	/	12	55%	29%	19%
		CCRT (36)					
Ma, D. Y., 2014^24^	98	3D-CRT alone (49)	73.5%	19	69.4%	/	28.6%
		CCRT with weekly cisplatin (49)	91.8%	35	85.7%		46.9%
Kobayashi, R., 2014^15^	42^c^	RT alone (7)	97.6%	24.3	81.2%	51.3%	41.1%
		CCRT with four-weekly regimen (35)					
Current study	27	CCRT with weekly CF (14)	70.4%	26	88.9%	60.2%	/
		CCRT with weekly NF (13)					

*Abbreviations:* 3D-CRT = three-dimensional conformal radiotherapy; CCRT = concurrent chemoradiotherapy; CF = cisplatin + 5-FU; CT = chemotherapy; MST = median survival time; NF = nedaplatin + 5-FU; OS = overall survival; ORR = overall response rate; RT = radiotherapy; TP = paclitaxel + cisplatin.

^a^Including seven patients having distant metastasis with (1 patient) or without locoregional recurrence (6 patients).

^b^Including 17 patients with adenocarcinoma and eight patients who received definitive radiochemotherapy as initial treatment.

^c^Including one patient with carcinosarcoma.
